# Optimizing Common Bean Symbiosis via Stage-Specific Reinoculation and Co-Inoculation

**DOI:** 10.3390/plants14172676

**Published:** 2025-08-27

**Authors:** Tamires Ester Peixoto Bravo, Itamar Rosa Teixeira, Gisele Carneiro da Silva Teixeira, Nathan Mickael de Bessa Cunha, Ednaldo Cândido Rocha, Lucas Boaretto Comachio, Gessiele Pinheiro da Conceição Alves

**Affiliations:** 1 Institute of Agricultural Science, State University of Goiás, Anápolis 75132-903, GO, Brazil; tamires.bravo@aluno.ueg.br (T.E.P.B.); gisele.carneiro@ueg.br (G.C.d.S.T.); nathan.cunha@aluno.ueg.br (N.M.d.B.C.); ednaldo.rocha@ueg.br (E.C.R.); 2 Department of Agricultural Engineering, Federal University of Viçosa, Viçosa 36570-900, MG, Brazil; lucas.comachio@ufv.br; 3 Department of Agronomy, Federal University of Goiás, Goiânia 74690-900, GO, Brazil

**Keywords:** *P. vulgaris*, plant nutrition, BNF, biomass accumulation, performance

## Abstract

The common bean relies on biological nitrogen fixation to meet part of its nitrogen requirements. This study aimed to evaluate the effect of reinoculation with *Rhizobium tropici*, alone or combined with *Azospirillum brasilense*, at different phenological stages. The experiments were conducted in the winter of 2023 and the rainy season of 2023/24, and significant differences were observed between seasons, mainly due to temperature and water stress, which impacted nodulation, plant growth and grain yield. However, appropriate water management mitigated these limitations, allowing reinoculation combined with co-inoculation at the V4 stage to improve nodular and morphophysiological traits, ensuring adequate nutrition through biological nitrogen fixation. This strategy promoted nodulation and plant development, resulting in an 8.5% increase in yield compared to nitrogen fertilization (80 kg ha^−1^), reaching 2197.87 kg ha^−1^. These results suggest that reinoculation with co-inoculation at the V4 stage can enhance biological nitrogen fixation, reduce dependence on synthetic fertilizers and serve as a sustainable and economically viable alternative.

## 1. Introduction

The common bean (*Phaseolus vulgaris* L.), a member of the Fabaceae family, plays a vital role in global food security. It is considered a significant source of plant-based protein and income for farmers, particularly in developing countries in Central and South America, Africa and Asia [1,2,3]. The global area cultivated with beans—which also includes cowpea (*Vigna unguiculata* (L.))—covers approximately 36 million hectares with an annual production of about 28,000 kg and an average yield of 700 kg ha^−1^ [4].

Brazil, the world’s largest bean producer, cultivates approximately 2.856 million ha, resulting in a production of 3.2 million and an average yield of 1100 kg ha^−1^ [5]. Although this performance remains below the crop’s productive potential, recent studies indicate that yields exceeding 3000 kg ha^−1^ are technically feasible [6,7,8]. Achieving these levels depends on using genetically improved cultivars, adopting efficient agronomic practices and utilizing favorable climatic conditions.

For common bean productivity, the main constraint is nutrient deficiency, particularly nitrogen (N), which is essential for plant growth. The crop generally requires between 100 and 140 kg ha^−1^ of nitrogen, which is also the most exported nutrient in harvested grains, averaging 33 kg of N per t of beans [6]. Producers typically use mineral fertilizers, such as urea or ammonium sulfate. However, the high cost of these inputs, coupled with environmental concerns, including greenhouse gas emissions and water contamination, has driven the search for more sustainable alternatives [9,10].

One sustainable alternative is biological nitrogen fixation (BNF), a natural process in which soil bacteria convert atmospheric N_2_ into plant-available forms, particularly in legumes [11]. The symbiosis between Rhizobium and legumes induces the formation of root nodules, structures where the bacteria colonize and reduce atmospheric nitrogen to ammonium (NH_4_^+^) via the nitrogenase enzyme complex. The fixed nitrogen in the form of NH_4_^+^ is readily available for plant assimilation, while the plant, in return, provides nutrients and a protected habitat for the microsymbiont, shielding it from biotic and abiotic stresses [12,13].

Several factors influence symbiosis in common bean, including the cultivar’s nodulation capacity, susceptibility to fungal diseases such as Fusarium root rot, climatic conditions, soil properties (clay content, organic matter, moisture and pH) and agricultural management practices, such as crop rotation and the application of fertilizers and agrochemicals [14,15,16]. A particularly relevant aspect for inoculation effectiveness in this crop is the occupation of nodules by competitive yet inefficient native rhizobia, which substantially reduces the contribution of BNF, considered the lowest among grain legumes [17].

In Brazil, *Rhizobium tropici* CIAT899 and *Rhizobium freirei* PRF81 are officially recommended for producing commercial inoculants for common bean [15,18]. These strains exhibit high BNF rates and strong competitiveness in nodulation. In this context, programs aimed at the continuous selection of more competitive, efficient, adapted, genetically stable and abiotic-stress-tolerant bacterial strains have the potential to optimize symbiosis, resulting in improved plant growth and higher grain yield [16].

Biotechnological advances have enabled the development of *Rhizobium tropici* strains, such as SEMIA 4077, SEMIA 4080 and SEMIA 4088, which show greater tolerance to environmental stresses related to soil acidity and high temperatures than conventional strains, making them more suitable for bean cultivation under diverse environmental conditions [19,20]. Moreover, these strains can help reduce both the economic and environmental costs of common bean production by partially replacing the use of nitrogen fertilizers [10,21].

With the use of BNF-selected strains, the average economic savings per hectare through inoculation increased from USD80 in 2006 to USD112 in 2017 [22]. However, despite these promising results, many farmers remain reluctant to adopt inoculation technologies when aiming for yields above 1800 kg ha^−1^. This highlights the need to improve inoculation strategies to make them more efficient and appealing to producers.

Among the studied alternatives, reinoculation during crop development stands out. This technique involves the supplemental application of *R. tropici* directly to the root zone during the crop cycle. It aims to maintain an active rhizobia population, ensuring a continuous nitrogen supply throughout plant development [23,24]. Evidence indicates that this practice can enhance nodulation and dry biomass accumulation, as well as supporting the formation of new functional nodules, particularly during the pod-filling stage [25,26].

Another promising strategy is co-inoculation with *Azospirillum brasilense*, a plant-growth-promoting bacterium that primarily functions through the production of phytohormones, such as auxins and cytokinins. These compounds stimulate root development, improve water and nutrient uptake and facilitate symbiosis with *R. tropici* [17,27,28]. Studies suggest that this synergistic interaction can increase nodulation, root growth, N acquisition [29,30] and bean yield, as well as seed physiological quality [31].

However, most research has focused on seed-applied inoculants [32], whereas studies on the topdressing application of inoculants remain limited to a single plant developmental stage [33], without many answers regarding the synergism of bacteria reapplied during plant development [34]. Understanding the responses of nodular, morphophysiological and agronomic traits of common bean plants to reinoculation at different phenological stages is of paramount importance for the efficiency of this technique.

Given this context, it is essential to investigate the effect of applying *R. tropici*, alone or in combination with *A. brasilense*, via topdressing at different developmental stages of the common bean crop. Furthermore, determining the optimal timing for such an application is crucial to maximizing BNF efficiency and contributing to a sustainable increase in crop productivity.

## 2. Results

### 2.1. Nodular Analyses

**Figure 1:**
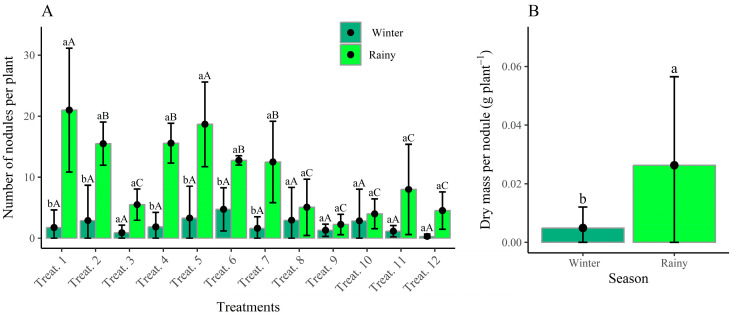
Number of nodules per plant (NNP) (**A**) as a function of treatments and dry mass per nodule (NDM) during the winter (2023) and rainy (2023/2024) growing seasons (**B**). Treat 1—Reinoculation in topdressing at V3, Treat 2—Reinoculation in topdressing at V4, Treat 3—Reinoculation in topdressing at R5, Treat 4—Reinoculation in topdressing at R6, Treat 5—Co-inoculation in topdressing at V3, Treat 6—Co-inoculation in topdressing at V4, Treat 7—Co-inoculation in topdressing at R5, Treat 8—Co-inoculation in topdressing at R6, Treat 9—Control, Treat 10—Inoculation via seed, Treat 11—Co-inoculation via seed, Treat 12—Nitrogen fertilization. Means followed by the same lowercase letter within each season do not differ significantly according to the Scott–Knott test (*p* ≤ 0.05). Means followed by the same uppercase letter and color between seasons for the same treatment do not differ significantly according to the Scott–Knott test (*p* ≤ 0.05).

Figure 1 presents the nodulation characteristics of common bean plants cultivated during different growing seasons. The treatments evaluated encompass co-inoculation and reinoculation strategies applied at various phenological stages, mineral nitrogen fertilization and a negative control treatment without fertilization.

**Figure 2:**
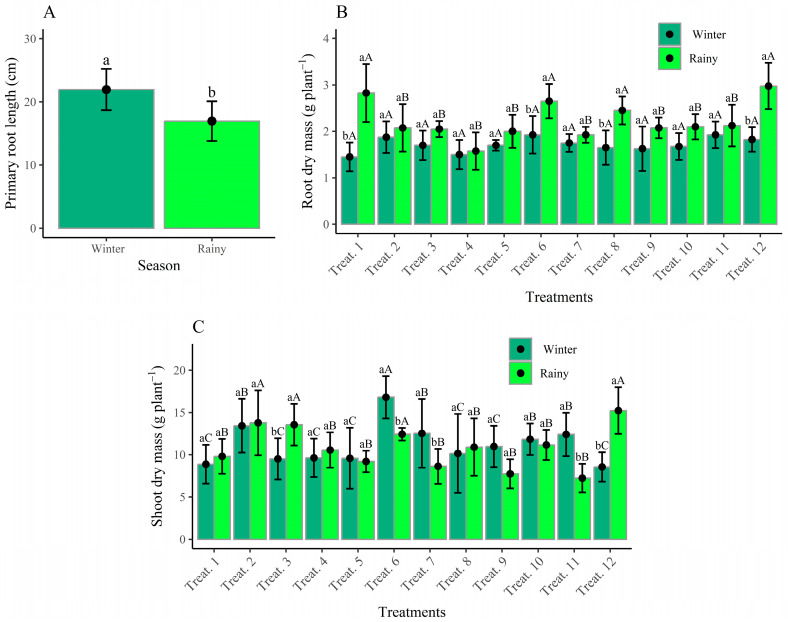
Primary root length as a function of growing seasons (**A**), root dry mass per plant (**B**) and shoot dry mass (**C**) as a function of treatments. Means followed by the same lowercase letter within each season do not differ significantly (Scott–Knott test, *p* ≤ 0.05). Means followed by the same uppercase letter and color between seasons for the same treatment do not differ significantly (Scott–Knott test, *p* ≤ 0.05).

Beans grown in the winter season showed low nodulation capacity, with an average of 2.8 nodules per plant. The treatment with the best result was T6 (co-inoculation via seed + reinoculation and co-inoculation at the V4 stage), with 5 nodules per plant (Figure 2A), which is considered low compared to the nodulation evaluation of bean plants cultivated during the rainy season. In this case, T1 stood out, receiving reinoculation of *R. tropici* at the V3 stage, with 22 nodules per plant (NNP), followed by T5 (co-inoculation via seed + reinoculation and co-inoculation at the V3 stage). Conversely, the lowest NNP values were observed in T9, which corresponded to the control treatment with no inoculant and no mineral fertilization, and in T12, which involved mineral fertilization alone, both treatments maintaining low performance across both growing seasons.

The results for dry mass per nodule (NDM) (Figure 2B) partially reflect the NNP data, as a significant effect was detected only between growing seasons, with emphasis on the rainy season, in which 0.0264 g of nodule dry mass per plant was obtained, corresponding to 26.4 mg of nodule dry mass per plant.

### 2.2. Morphological Analyses

Bean plants cultivated during the winter 2023 season exhibited greater primary root length (PRL) (Figure 2A), with an average value of 22 cm, representing 29.4% greater vertical growth compared to the rainy season (2023/2024), which recorded a PRL of 17 cm.

The results for primary root length (PRL) demonstrate superior performance during the winter season, differing statistically from the rainy season (Figure 2A). This contrasts with the results obtained for root dry mass. For root dry mass per plant, an interaction effect between growing seasons and treatments was observed, with 22.7% greater performance in the rainy season compared to the winter season (Figure 2B). The treatments that resulted in the highest root system dry mass were T12 (nitrogen fertilization) and T6 (reinoculation + co-inoculation at V4) in both growing seasons. Additionally, T1 (reinoculation at V3) stood out with a root dry mass of 2.825 g per plant in the rainy season.

The aerial part dry mass (APDM) of bean plants was significantly affected by the interaction between growing seasons and treatments (Figure 2C). In the winter season, Treatment 6, which received seed co-inoculation combined with topdressing at the V4 stage, achieved the highest APDM (16.8 g plant^−1^). In the rainy season, Treatments 2 and 3 reached 15.6 g plant^−1^ when reinoculated via topdressing with *R. tropici* at the V4 and R5 stages, respectively. Treatment 12, involving nitrogen fertilization, recorded an APDM of 14.9 g plant^−1^. Regarding leaf nitrogen content (LNC), the average values ranged from 35 to 41 g kg^−1^, with no significant differences between growing seasons, treatments or the interaction between factors.

### 2.3. Agronomic Analyses

**Figure 3:**
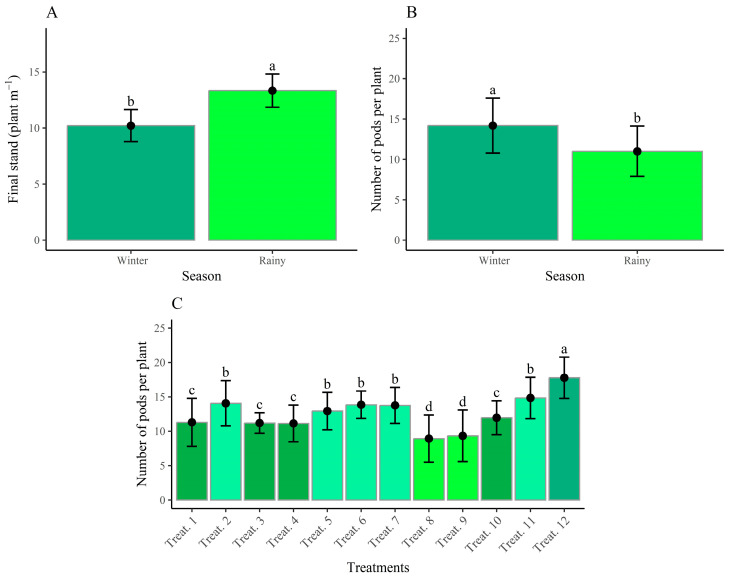
Final stand (**A**) and number of pods per plant (**B**) as a function of growing seasons and number of pods per plant (**C**) as a function of treatments in the combined analysis of growing seasons. Means followed by the same letter and color do not differ according to the Scott–Knott test at the 5% probability level.

Figure 3 presents the agronomic performance parameters of the evaluated treatments. The final plant stand (FPS) was influenced exclusively by the growing seasons (Figure 3A), with averages ranging from 11 plants per linear meter in the winter 2023 season to 13 plants per linear meter in the rainy 2023/2024 season, values that did not compromise productivity due to the compensatory capacity of the bean plants.

The number of pods per plant (NPP) also showed a significant difference between growing seasons (Figure 3B), with the winter 2023 season performing better, averaging 15 pods per plant, corresponding to a 21.4% increase over the rainy 2023/2024 season (Figure 3C). This variable was also responsive to the treatments analyzed. The highest NPP was observed in treatment T12, which received mineral fertilization, followed by the co-inoculated treatment T11, applied via seed and reinoculated via topdressing at V3, V4 and R5, respectively, and treatments T5, T6 and T7. Treatment T1, reinoculated with *R. tropici* at V4, was also included in this group. The lowest results were observed in the control (T9) and T8—co-inoculation at R6, which did not differ statistically from each other.

**Figure 4:**
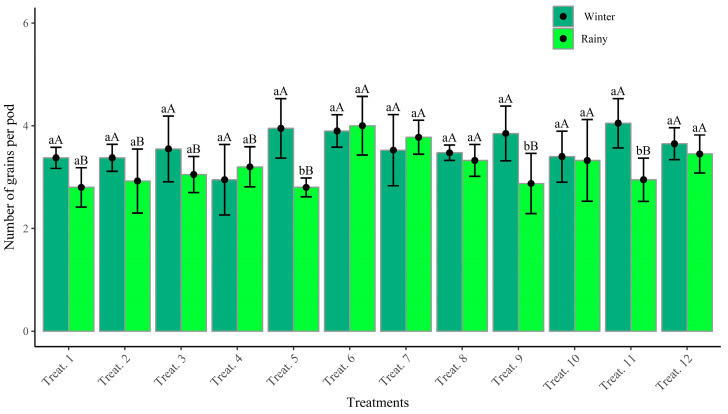
Number of grains per pod as a function of treatments. Means followed by the same lowercase letter within each season do not differ significantly according to the Scott–Knott test (*p* ≤ 0.05). Means followed by the same uppercase letter and color between seasons for the same treatment do not differ significantly according to the Scott–Knott test (*p* ≤ 0.05).

The values for the number of grains per pod (NGP) ranged from 3 to 4 grains per pod in the winter season and from 2 to 4 grains per pod in the rainy season. The highest averages were observed in the co-inoculated treatments, followed by nitrogen mineral fertilization (Figure 4). Notably, there was consistency in the results for treatments T6 (co-inoculation at V4) and T7 (co-inoculation at R5), which showed good performance in both growing seasons, as well as in the nodular and morphophysiological characteristics evaluated in this study, thus corroborating the relationship between the analyzed components. Treatment 6 presented the best performance in both growing seasons, numerically surpassing treatment 12, which received mineral fertilization.

**Figure 5:**
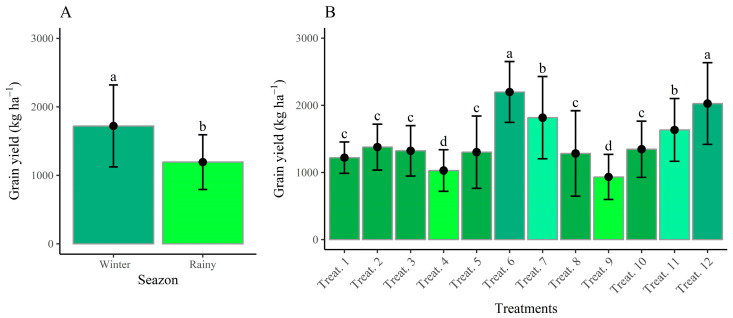
Grain yield as affected by growing seasons (**A**) and treatments (**B**). Means followed by the same letter and color do not differ according to the Scott–Knott test (*p* ≤ 0.05).

Grain productivity during the rainy season was 27.16% lower than that in the winter season (Figure 5A). This difference can be attributed to the greater water availability during the winter season, which was irrigated, thereby preventing water stress and flower abortion. In contrast, the rainy season relied exclusively on precipitation, thus limiting water availability. Grain yield was also influenced by the various treatments applied (Figure 5B).

The reinoculation technique—consisting of co-inoculating *Rhizobium tropici* and *Azospirillum brasilense* at the V4 growth stage (T6)—as well as nitrogen mineral fertilization applied both at planting and as a topdressing (T12) resulted in yields of 2197.87 kg ha^−1^ and 2025.75 kg ha^−1^, respectively. These values were significantly higher than those observed for the other treatments. Conversely, the control treatment (T9), which received no nitrogen input, recorded the lowest yield, at only 932.75 kg ha^−1^.

### 2.4. Principal Component Analysis

**Figure 6:**
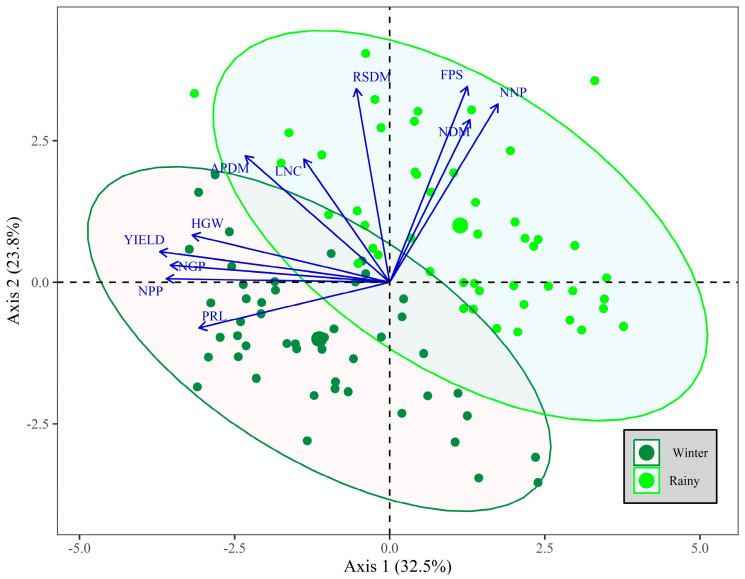
Principal component analysis (PCA) map of the nodular, morphophysiological and agronomic characteristics of bean plants grown in the winter (2023) and rainy (2023/2024) seasons.

The results of the principal component analysis (PCA) of the variables analyzed in this study were plotted on a two-dimensional diagram (Figure 6), explaining 56.3% of the total variation, with 23.8% on axis 1 and 32.5% on axis 2. Data were classified according to the growing season: winter 2023 or rainy season 2023/2024. Additionally, to assess similarity, confidence ellipses were included around the data.

**Figure 7:**
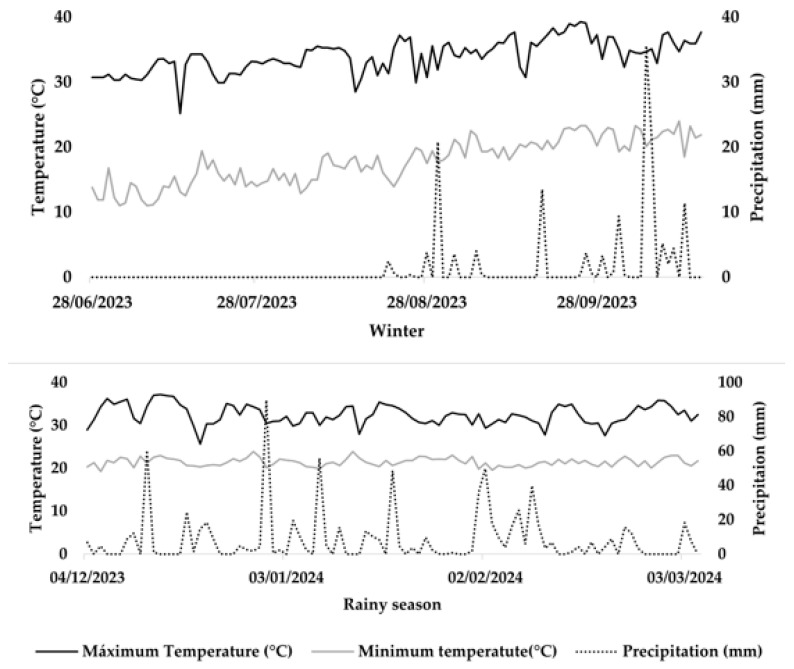
Climate data referring to maximum and minimum temperatures and precipitation in the experimental period, from July 2023 to March 2024, in Anápolis-GO municipality, Brazil.

Following the results of the nodular and morphophysiological analyses conducted at R7, the PCA (Figure 6) indicated superior performance for these characteristics in the rainy season, during which the bean plants were subjected to optimal temperature and humidity conditions throughout their vegetative period. Conversely, the winter season showed limited responsiveness in the nodular and morphophysiological evaluations due to thermal accumulation during its early stages. However, favorable temperature conditions (Figure 7) during the reproductive period and adequate water availability provided by irrigation resulted in superior yields compared to the rainy season. The minimal overlap between the confidence ellipses confirms this relationship.

By analyzing the behavior of the variables, it is possible to validate this hypothesis. The agronomic variables, such as yield (YIELD), number of pods per plant (NPP) and number of grains per pod (NGP), exhibit similar trends, with consistent growth patterns and standardized orientations, confirming their contribution and relationship. In contrast, the nodular characteristics (NNP and NDM) tend to show opposite trends compared to agronomic ones, demonstrating little correlation, as indicated by the low overlap between the confidence ellipses. However, it is important to emphasize that PCA is an exploratory analysis, and theoretical knowledge regarding the subject under study should guide the conclusions.

It is also evident that the variable’s aerial part dry mass (APDM) and leaf nitrogen content (LNC) show similar behaviors in terms of variation levels, with substantial overlap of the confidence ellipses. This indicates that the inoculant-based treatments ensured nitrogen nutrition compared to the control in challenging conditions faced during different growing seasons. The results were even more favorable when considering the yields of the co-inoculated treatments.

## 3. Discussion

Overall, nodulation in plants grown during the winter season was less efficient than in the rainy season. This reduction can be attributed to low temperatures (around 15 °C; Figure 7) during the early stages of the crop, which delayed seed germination and vegetative development, thus affecting *R. tropici* infection and increasing competition with native soil bacteria that are more tolerant to adverse conditions [35]. Previous studies indicate that the optimal temperature range for rhizobia to perform biological nitrogen fixation (BNF) in association with beans is between 25 °C and 38 °C [13,15] which is higher than the maximum and minimum temperatures recorded in the winter crop (approximately 30 °C to 15 °C; Figure 7).

The significant interaction between the growing season and treatments for the number of nodules per plant (NNP) highlights how this variable responded differently to treatments across seasons. The highest NNP value (22) was observed during the rainy season in treatment T1 (reinoculation at stage V3), which was statistically similar to T5 (co-inoculation at V3) (Figure 1A). This value is close to the one reported by [33], which observed 24 NNP in the *BRS Esteio* cultivar co-inoculated with *R. tropici* + *A. Brasiliense* at stage V4.

Nodule dry mass per plant (NDM) results indicated a significant difference between planting seasons, with the rainy season standing out with the best result. Although NDM was not influenced by the treatments studied, it was possible to observe that the use of co-inoculation via seed and topdressing enabled adequate nodulation, reaching average values above 20 NNP and NDM greater than 100 mg per plant, which are considered satisfactory for good BNF efficiency according to [36]. Numerically, inoculation and co-inoculation treatments consistently outperformed nitrogen topdressing for both NNP and NDM, supporting literature reports of the negative effect of mineral nitrogen on rhizobacterial activity [24].

In winter, although low temperatures limited initial nodulation, the availability of irrigation technology and temperatures above 25 °C during the reproductive stage promoted plant growth, including root development, as shown by the primary root length (PRL) analysis result (Figure 2A). A larger root system increases root–soil contact, improving nutrient uptake and yield potential [37].

The differences between the evaluated seasons reflect the inherent variability of agricultural activities, often influenced by uncontrollable factors, such as atmospheric conditions. In this study, the winter crop was impacted by abiotic factors, such as thermal accumulation associated with low temperatures during the early stages of development, resulting in a reduction in the final plant stand (FPS). However, this effect contributed to a higher number of pods per plant compared to the rainy season crop due to the physiological compensatory capacity of the common beans [38]. In contrast, the rainy season crop, which did not face the same stress, maintained a full stand but with a smaller compensatory response.

This study found no significant differences in leaf nitrogen content between treatments. This lack of significance is attributed to the translocation of nitrogen from the leaves to the developing grains during the R7 stage (pod formation), creating a dilution effect that masks the differences between treatments [39]. Similar results were reported by [6,32], which found no significant influence of leaf nitrogen content in bean plants subjected to inoculation treatments.

The increase in aerial part of dry mass (APDM) observed with co-inoculation is likely linked to *A. brasilense*, which stimulates root growth through the production of auxin, cytokines and gibberellins [27,28]. These phytohormones promote root expansion and, when combined with *R. tropici*, increase nodulation [19]. In this context, treatment T6 produced 50.1% more SDM than the control in response to seed co-inoculation with *A. brasilense*, followed by reinoculation at the V4 stage.

The success of the reinoculation technique depends on choosing the ideal stage for reapplication of rhizobacteria. At the V4 stage, the plant prepares for reproduction and requires high nutritional requirements, particularly nitrogen (N). The availability of rhizobacteria through complementary application enhanced the nodulation process and, consequently, BNF, promoting plant nutrition.

Similarly, ref. [33], examining the effects of co-inoculation and reinoculation at different doses at the V4 stage for the cultivar *BRS Estilo*, reported that the presence of rhizobia inoculants combined with *A. brasilense* improved root system development, with an average length of 20.4 cm. They also found a 115% increase in root system dry mass (RSDM) and a 65% increase in aerial part of dry mass (APDM) compared to the control. Furthermore, RSDM and APDM showed similar responses to the treatments, indicating a strong relationship between these traits.

Considering the agronomic results, the treatments with mineral fertilizer (T12) and co-inoculation at V4 (T6) stand out. This confirms the efficiency of the technique involving co-inoculation via seed followed by reinoculation at the V4 stage, as it provides good vegetative development with nutritional demands met viabiological nitrogen fixation (BNF). These findings are consistent with the research by [33,40], who evaluated the production components and productivity of common beans when inoculated with *R. tropici* and/or *A. brasilense*. They observed that reinoculation and co-inoculation promoted an increase in the number of pods per plant. The use of this technique presented productivity results similar to those obtained with mineral nitrogen fertilization, confirming the efficiency of the process.

Additionally, the highest average productivity (YIELD) recorded in this study for treatment T6 (2197 kg ha^−1^) surpassed the national average productivity of the 2023/2024 harvest, which was 1138 kg ha^−1^ [5]. These results highlight the benefits of co-inoculation at the V4 stage, which proved to be an efficient technique to increase production more sustainably and economically, corroborating the reports of [22] on the cost-effectiveness generated by the use of inoculation over the use of nitrogen fertilizers. In addition to being more affordable than mineral fertilizers, the use of inoculants helps reduce the use of chemical inputs, whose production cycle is highly polluting [41], which supports the practice’s environmental viability in the management of common beans.

In economic terms, the investment required for inoculants based on *Rhizobium tropici* ranges from approximately $ 1.54 to $ 2.88 ha^−1^, whereas conventional nitrogen fertilization with urea exceeds $ 42.31 ha^−1^ [42], clearly demonstrating the significant cost reduction provided by this biological technology. The co-inoculation of *R. tropici* and *A. brasilense* in different soil types found that sandy soil produced the largest profit, with the highest yield reported in sandy soil, which was 25.6% higher than that obtained with mineral nitrogen fertilizer [21]. In this scenario, the production cost with mineral fertilizer was $ 518.79 ha^−1^ compared to $ 414.83 ha^−1^ for co-inoculation.

From an environmental perspective, the use of inoculants reduces the dependence on chemical inputs, whose production cycle is highly polluting, while also mitigating greenhouse gas emissions associated with the manufacture and application of mineral fertilizers [10,40]. Furthermore, this sustainable approach promotes soil health by stimulating beneficial microbiota, contributing to greater resilience of production systems and aligning with the principles of ecological intensification in agriculture [43].

The results obtained from principal component analysis (PCA) indicate that delayed vegetative development was not as significant a factor in productivity as water stress during the reproductive phase. According to [44], water scarcity during the reproductive stages reduces photosynthesis and decreases grain filling efficiency, negatively impacting productivity. Previous studies also reported a 60–99% reduction in common bean productivity when exposed to water stress during the flowering and post-flowering phases [45], values higher than those found in this study.

The number of pods per plant (NPP) and the number of grains per pod (NGP) are factors directly linked to productivity. In addition to being genotypic characteristics of the cultivar, they can also be associated with management practices and environmental factors [44], such as the reduction in the final stand (FPS). Although temperature and water availability are determining factors for productivity, plant nutrition is essential, with nitrogen being highly demanded by common beans [46]. Its deficiency results in low productivity levels, as observed in the control treatment (T9), which produced only 932.75 kg ha^−1^ of bean grains.

Based on the PCA results, it can be concluded that grain yield was affected by the number of pods per plant and the number of grains per pod, which were influenced by the growing seasons. This caused the differentiated behavior of the *BRS Estilo* cultivar, explained by temperature and water availability. It is also evident that the variables of aerial part of dry mass (APDM) and leaf nitrogen content (LNC) showed similar patterns of variation, with good overlap of the confidence ellipses. This behavior indicates that the inoculant-based treatments provided nitrogen nutrition comparable to the control, regardless of the challenging conditions in different growing seasons. The results were even more favorable when considering the yields of the co-inoculated treatments (Figure 5B).

Therefore, based on the yield obtained for treatment T6, which received reinoculation at the V4 stage with co-inoculation of *R. tropici* and *A. brasilense*, it can be concluded that the nitrogen needs of the common bean crop were effectively met through biological nitrogen fixation (BNF) using the methods applied. However, the behavior of nodular, morphophysiological and agronomic variables across different bean cultivation seasons showed that abiotic factors significantly influence these variables. For example, low temperatures during the early stages of plant growth and drought conditions during flowering were observed, with water stress emerging as a major limiting factor in the productivity of common beans.

It is suggested that future studies evaluate the nodulation capacity of common bean in response to rhizobacterial inoculants under different temperature and moisture conditions in order to understand how abiotic stress influences the process of biological nitrogen fixation and crop development. Such investigations would support the refinement of management recommendations and maximize productive efficiency.

## 4. Materials and Methods

### 4.1. Experimental Field Characterization

The experiment was carried out in two crop seasons: in “winter” (2023) and the “rainy season” (2023/2024), in two 600 m^2^ plots, in the experimental area of Agência Goiânia de Assistência Técnica, Extensão Rural e Pesquisa Agropecuária–Emater, located in the municipality of Anápolis-GO, Brazil, (16°20′12.13″ S; 48°53′15.96″ W; mean altitude: 1058 m). The region’s weather, according to the Köppen classification, is Aw (tropical savanna climate), with an annual mean temperature of 22 °C and an annual mean precipitation of 1677 mm [47]. The climate data from the experiment conducted are presented in Figure 7.

The experimental area’s soil was classified as Oxisols [48] and was previously cultivated with corn (*Zea mays*). For its physical–chemical characterization, samples were taken from 0 to 20 cm and sent for analysis in a specialized laboratory. For the winter season, the soil analysis results showed pH (CaCl_2_) 5.1, P (mg dm^−3^) 18.1, K (cmol_c_dm^−3^) 0.36, Ca (cmol_c_dm^−3^) 2.9, Mg (cmol_c_dm^−3^) 1.0, Al (cmol_c_dm^−3^) 0, H + Al (cmol_c_dm^−3^) 3.8, base saturation (%) 52.9, B (mg dm^−3^) 0.0, Cu (mg dm^−3^) = 2.7, Fe (mg dm^−3^) 19.8, Mn (mg dm^−3^) 22.8, Zn (mg dm^−3^) 11.1, organic matter (g dm^−3^) 31.0, sand (g kg^−1^) 450, silt (g kg^−1^) 110 and clay (g kg^−1^) 440. Meanwhile, for the rainy season, the analysis presented pH (CaCl_2_) 5.0, P (mg dm^−3^) 12.7, K (cmol_c_dm^−3^) 0.31, Ca (cmol_c_dm^−3^) 2.8, Mg (cmol_c_dm^−3^) 0.7, Al (cmol_c_dm^−3^) 0, H + Al (cmol_c_dm^−3^) 2.9, base saturation (%) 56.94, B (mg dm^−3^) 0.0, Cu (mg dm^−3^) 2.2, Fe (mg dm^−3^) 26.2, Mn (mg dm^−3^) 19.3, Zn (mg dm^−3^) 6.5, organic matter (g dm^−3^) 28.0, sand (g kg^−1^) 450, silt (g kg^−1^) 110 and clay (g kg^−1^) 440.

According to the results, soil analysis indicated the need for liming for both plots. Application of 2.0 t ha^−1^ of dolomitic limestone was performed to correct the soil’s high acidity and aimed to reach 70% base saturation. Planting was postponed for three months to allow the liming reaction to occur.

### 4.2. Experimental Design and Treatments

The randomized block experimental design was used with 4replications and 12treatments, for a total of 48 experimental parcels. The treatment’s tests are described in Table 1. Reinoculated treatments were initially subjected to application of commercial liquid inoculants through seeds, containing cells of *R. tropici* (SEMIA4088 and SEMIA 4077) (2 × 10^9^ CFU mL^−1^), at a dose of 150 mL for every50 kg of seeds and co-inoculation with *A. brasilense* (3 × 10^9^ UFC mL^−1^) at a dose of 100 mL per 50 kg of seeds, as recommended by the manufacturers for bean crops. Later, those treatments received reinoculations in different phenological development phases, with triplicate doses, in order to potentiate the interactions between biological products and soil particles [33]. In addition, another four treatments were tested: control (without nitrogen fertilizers); inoculation with *R. tropici* through seeds; co-inoculation with *A. brasilense* through seeds; and mineral nitrogen fertilization doses of 16 and 64 kg of N per ha applied at base and topdressing, respectively.

### 4.3. Implementation and Management

Soil tillage was conducted by conventional means, with plowing followed by two harrows. Parcels were composed of four rows, 5 m long, and spaced 0.50 m from each other. Both central lines were considered usable areas, while both lateral lines and 0.50 cm from the parcel’s extremities served as borders. A density of 12 plants per linear meter was adopted. Common bean cultivar cv. *BRS Estilo* was used, which presents carioca-type grains, erect growth habit, indeterminate growth cycle (type II), a medium cycle of 90 days and adaptation to mechanized harvesting, with productivity potential of 4.0-t ha^−1^ [49].

For the base fertilization in all experimental parcels, 400 kg ha^−1^ of NPK 04-30-20 was applied in the planting furrows. Also, 16 kg ha^−1^ N was applied at the base of inoculation and/or co-inoculation treatments to supply the nutrients for initial plant growth, as the inoculated bacteria are still not fully active in the bean’s root system [24].

Subsequently, the reinoculation procedures were initiated at the V3 stage and completed at the R6 stage, with applications performed using a 5L sprayer, with a jet directed toward the soil to deliver the bacterial populations to the root system of the plants. The topdressing fertilization was carried out only in the treatment with nitrogen mineral fertilizer, using urea as the nitrogen source at a rate of 80 kg ha^−1^. This dose was split into two applications, at 25 and 35 DAE, to minimize losses due to volatilization and/or leaching.

In the winter crop, sprinkler irrigation was applied throughout the entire bean growth cycle to meet the plants’ water requirements. Irrigation was scheduled twice per week during the vegetative growth stage and three times per week after flowering and during grain filling. In contrast, during the rainy season, water requirements were met exclusively through precipitation.

### 4.4. Evaluated Parameters

At the R7 stage (full pod filling), five whole plants were sampled from the first 0.50 m of the two central rows of each plot using a flat spade, recovering the root system to a depth of 0–0.30 m. Roots and shoots were washed in running water, and plants were sectioned at the collar for separate evaluation of root and shoot traits, focusing on nodular and morphophysiological characteristics.

Nodular traits included the number of nodules per plant (NNP; nodules > 1 mm), expressed as the mean of five plants, and nodule dry mass (NDM), determined after oven-drying at 65 °C for 72 h and weighing to the nearest 0.001 g. NDM per plant was calculated as the ratio between dry mass and nodule number [50].

The morphophysiological traits evaluated comprised (a) primary root length (PRL), measured from the plant collar to the root tip using a ruler, with values expressed as the arithmetic mean of five sampled plants in centimeters; (b) root system dry mass (RSDM) and aerial part dry mass (APDM), determined after separating plant organs into roots and shoots, placing samples in kraft paper bags, oven-drying in a forced-air circulation oven at 72 ± 1 °C for 72 h and weighing on an analytical balance (0.001 g), with results expressed in grams per plant [33]; and (c) leaf nitrogen content (LNC), determined using the macro-Kjeldahl method, as described in ref. [48].

At the R9 stage (full maturity), agronomic traits were assessed. The final plant stand (FPS) was determined by counting all plants in the useful plot area. Ten plants were sampled for yield component evaluation: number of pods per plant (NPP), number of grains per pod (NGP) and hundred-grain mass (HGM). Grain yield (YIELD) was obtained from all plants in the useful area, weighed to the nearest 0.01 g, corrected to 13% moisture and expressed in kg ha^−1^.

### 4.5. Statistical Analysis

Data were tested for residual normality and variance homogeneity, followed by ANOVA and the Scott–Knott test (5% probability), in a joint analysis of both growing seasons, using the ExpDes.pt package [51] in RStudio v. 2022. Graphs were produced with ggplot2 v. 3.5.2 [52], and principal component analysis (PCA) was performed with the factoextra package v. 1.0.7 [53].

## 5. Conclusions

The results demonstrate that the application of liquid inoculant as a topdressing at the V4 stage improved the nodular and morphophysiological traits of common bean plants, ensuring adequate nutrition through biological nitrogen fixation.

The environmental conditions significantly influenced plant performance in both cropping seasons. Although thermal accumulation during the early vegetative stages negatively affected nodulation and morphophysiological characteristics in the winter crop, controlled irrigation during the reproductive phase compensated for these limitations, resulting in higher yields. In contrast, despite favorable vegetative conditions, the rainy season crop experienced water stress during the reproductive phase, which ultimately reduced yield. The use of irrigation technology for water management, combined with optimal temperature conditions during the reproductive phase, can overcome delays caused by early thermal stress and ensure satisfactory yield levels.

The reinoculation technique combining *R. tropici* and *A. brasilense* in co-inoculation at the V4 stage resulted in an 8.5% increase in grain yield compared with conventional nitrogen fertilization, demonstrating its potential as a viable, sustainable and economically accessible alternative.

## Figures and Tables

**Table 1:** Treatments description of winter 2023 and rainy season 2023/2024 experiments.

Treatment	Description
T1	Reinoculation with *R. tropici* topdressing at V3
T2	Reinoculation with *R. tropici* topdressing at V4
T3	Reinoculation with *R. tropici* topdressing at R5
T4	Reinoculation with *R. tropici* topdressing at R6
T5	Co-inoculation with *R. tropici* + *A. brasilense* topdressing at V3
T6	Co-inoculation with *R. tropici* + *A. brasilense* topdressing at V4
T7	Co-inoculation with *R. tropici* + *A. brasilensis* topdressing at R5
T8	Co-inoculation with *R. tropici* + *A. brasilensis* topdressing at R6
T9	Control
T10	Inoculation with *R. tropici* via seeds
T11	Co-inoculation with *R. tropici* + *A. brasilense* via seeds
T12	Mineral nitrogen fertilization

## Data Availability

The original contributions presented in this study are included in the article. Further inquiries can be directed to the corresponding author.

## Abbreviations

The following abbreviations are used in this manuscript:

DAE	Days After Emergence
NNP	Number of Nodules per Plant
NDM	Nodule Dry Mass
PRL	Primary Root Length
RSDM	Root System Dry Mass
APDM	Aerial Part of Dry Mass
LNC	Leaf Nitrogen Content
FPS	Final Plant Stand
NPP	Number of Pods per Plant
NGP	Number of Grains per Pod
HGM	Hundred Grain Mass
YIELD	Grain Yield
